# PBPK‐led assessment of antimalarial drugs as candidates for Covid‐19: Simulating concentrations at the site of action to inform repurposing strategies

**DOI:** 10.1111/cts.13865

**Published:** 2024-07-17

**Authors:** Nada Abla, Lisa M. Almond, Jennifer J. Bonner, Naomi Richardson, Timothy N. C. Wells, Jörg J. Möhrle

**Affiliations:** ^1^ MMV Medicines for Malaria Venture Geneva Switzerland; ^2^ Certara UK Ltd Sheffield UK; ^3^ Magenta Communications Ltd Abingdon UK

## Abstract

The urgent need for safe, efficacious, and accessible drug treatments to treat coronavirus disease 2019 (COVID‐19) prompted a global effort to evaluate drug repurposing opportunities. Pyronaridine and amodiaquine are both components of approved antimalarials with in vitro activity against severe acute respiratory syndrome coronavirus 2 (SARS‐CoV‐2). In vitro activity does not always translate to clinical efficacy across a therapeutic dose range. This study applied available, verified, physiologically based pharmacokinetic (PBPK) models for pyronaridine, amodiaquine, and its active metabolite N‐desethylamodiaquine (DEAQ) to predict drug concentrations in lung tissue relative to plasma or blood in the default healthy virtual population. Lung exposures were compared to published data across the reported range of in vitro EC_50_ values against SARS‐CoV‐2. In the multicompartment permeability‐limited PBPK model, the predicted total *C*_max_ in lung mass for pyronaridine was 34.2 μM on Day 3, 30.5‐fold greater than in blood (1.12 μM) and for amodiaquine was 0.530 μM, 8.83‐fold greater than in plasma (0.060 μM). In the perfusion‐limited PBPK model, the DEAQ predicted total *C*_max_ on Day 3 in lung mass (30.2 μM) was 21.4‐fold greater than for plasma (1.41 μM). Based on the available in vitro data, predicted drug concentrations in lung tissue for pyronaridine and DEAQ, but not amodiaquine, appeared sufficient to inhibit SARS‐CoV‐2 replication. Simulations indicated standard dosing regimens of pyronaridine‐artesunate and artesunate‐amodiaquine have potential to treat COVID‐19. These findings informed repurposing strategies to select the most relevant compounds for clinical investigation in COVID‐19. Clinical data for model verification may become available from ongoing clinical studies.

**Study Highlights**

**WHAT IS THE CURRENT KNOWLEDGE ON THE TOPIC?**

Physiologically based pharmacokinetic (PBPK) modeling can be used to accelerate drug development by predicting drug concentrations in target tissues based on currently available pharmacokinetic data.

**WHAT QUESTION DID THIS STUDY ADDRESS?**

The study used PBPK modeling to assess whether the antimalarial combination therapies pyronaridine‐artesunate and artesunate‐amodiaquine could be repurposed for the treatment of COVID‐19.

**WHAT DOES THIS STUDY ADD TO OUR KNOWLEDGE?**

The study reports the predicted concentrations of pyronaridine, amodiaquine, and its metabolite DEAQ in the lung tissue and epithelial lining fluid of a healthy virtual population. These concentrations were then compared across the range of reported in vitro activity against SARS‐CoV‐2. The study supports the selection of pyronaridine and amodiaquine for clinical investigation in COVID‐19.

**HOW MIGHT THIS CHANGE CLINICAL PHARMACOLOGY OR TRANSLATIONAL SCIENCE?**

The study suggests that PBPK modeling can be a valuable tool for predicting drug concentrations in target tissues and informing the repurposing of existing drugs for new indications, such as treating COVID‐19. Additionally, the study highlights an approach to consider factors like changes in lung pH during COVID‐19 infection when optimizing drug therapies. Although clinical data are not yet available, the study encourages further exploration of the relationship between PBPK modeling and clinical outcomes in the development of drugs for infectious diseases.

## INTRODUCTION

The coronavirus disease 2019 (COVID‐19) pandemic, caused by severe acute respiratory syndrome coronavirus 2 (SARS‐CoV‐2), led to an estimated 14.9 million excess deaths between 2020 and 2021 as health systems became overwhelmed.^1^ Although vaccination programs have been successful in some countries, access remains an issue, and the risk of vaccine escape with the emergence of new variants persists.^2^

Despite extensive mobilization of resources, only a limited number of drugs have been approved for treating COVID‐19 for patients hospitalized with severe disease or in high‐risk populations in the community.^3^ However, medicines are required for wider community use in resource‐poor settings where hospital access is limited and patient risk may be unknown. This need was particularly acute at the height of the pandemic.

In early 2020, the antimalarial drugs pyronaridine and amodiaquine were identified as potential candidates for further investigation against COVID‐19.^4^, ^5^, ^6^, ^7^, ^8^, ^9^ These drugs have well known pharmacokinetic (PK), safety, and tolerability profiles, they are affordable and accessible and used widely in communities in malaria endemic countries. Because clinical data are already available, repurposing these drugs accelerates the development process, particularly if the approved dosing regimens for uncomplicated malaria are efficacious in COVID‐19.

Pyronaridine is a benzonaphthyridine derivative formulated as the tetraphosphate salt in a fixed‐dose combination with artesunate.^10^ Pyronaridine PK profiles have been established following single and multiple doses in healthy volunteers.^11^, ^12^ Pyronaridine antiviral activity was first demonstrated against Ebola and Marburg viruses.^13^ Further investigations through virtual screening revealed a high affinity between pyronaridine and the receptor binding domain of the SARS‐CoV‐2 spike protein, suggesting that its potential against the virus should be explored.^14^ Subsequent in vitro studies showed potent activity against SARS‐CoV‐2,^8^, ^9^, ^13^, ^15^ and pyronaridine exhibited protective effects against SARS‐Cov‐2 infection in mice.^16^ However, artesunate which is a component of the approved antimalarial formulation does not exhibit activity against SARS‐CoV‐2 in vitro.^17^

Amodiaquine, a 4‐aminoquinoline derivative, is formulated as a hydrochloride in a fixed‐dose combination with artesunate.^18^ The PK profiles of amodiaquine and its major metabolite N‐desethylamodiaquine (DEAQ) have been well described following single and multiple doses.^19^, ^20^, ^21^ Virtual and in vitro screening identified amodiaquine as a potential therapy for COVID‐19.^7^ Amodiaquine, as well as DEAQ, demonstrated activity against SARS‐CoV‐2 in human cells,^5^, ^6^, ^7^, ^8^, ^15^, ^22^, ^23^, ^24^, ^25^ and in the human‐airway‐on‐a‐chip model at clinically relevant doses.^22^

SARS‐CoV‐2 infection initially occurs in the upper respiratory tract but becomes established in the lung due to the high concentration of the target receptor, angiotensin‐converting enzyme 2 (ACE2) in lung tissue.^26^ Thus, achieving pharmacodynamically relevant drug concentrations in the lung is important in determining the therapeutic potential and appropriate dose of investigational COVID‐19 drug treatments. Pyronaridine and amodiaquine are basic amines and may be subject to lysosomal trapping, which could lead to accumulation in the lungs.^27^

Physiologically based pharmacokinetic (PBPK) modeling is a commonly used approach in drug development.^28^ Drug‐specific physicochemical, in vitro, and in vivo data are used to describe and predict drug concentrations in plasma and tissues across simulated populations. For drug repurposing, human PK data can be used to verify and refine the model. Verified models can then be combined with in vitro or in vivo activity assays to predict efficacious doses in patients for the new target indication.

The objective of this study was to use PBPK modeling to predict pyronaridine, amodiaquine, and DEAQ drug concentrations in the lungs following the administration of pyronaridine‐artesunate or artesunate‐amodiaquine at the approved dosing regimens for the treatment of uncomplicated malaria. The aim was to determine whether these predicted drug concentrations support the potential efficacy of these drugs in treating COVID‐19.

## METHODS

### PBPK model development and validation

The PBPK models for pyronaridine, amodiaquine, and DEAQ were developed using the Simcyp Simulator (Simcyp Ltd, Sheffield UK). Compound files and validation data have been previously published in full.^29^ Briefly, model input values for pyronaridine, amodiaquine, and DEAQ drug‐specific characteristics and PK parameters were obtained from the published literature, from in‐house data, or were predicted. All simulations were run using version 21 of the Simcyp Simulator. The PBPK models were validated against measured blood (pyronaridine) or plasma (amodiaquine and DEAQ) concentrations from clinical trials in healthy volunteers.^29^ Concentration–time profiles for pyronaridine and amodiaquine/DEAQ were generated using the Simcyp ‘healthy volunteer’ virtual population.^29^

### PBPK lung models

The PBPK models were used to estimate the lung concentration–time profiles as well as the ratio of blood (pyronaridine) or plasma (amodiaquine and DEAQ) to lung tissue concentrations. All simulations assumed that both pyronaridine‐artesunate and artesunate‐amodiaquine were dosed according to their approved doses for the treatment of uncomplicated malaria in adults, that is, once daily for 3 days at a daily dose of pyronaridine free base of 410 mg, corresponding to four tablets of 180‐mg pyronaridine tetraphosphate and 60‐mg of artesunate, and amodiaquine free base 540 mg, corresponding to two tablets of 270 mg (base) formulated as the hydrochloride salt and 100‐mg artesunate.

The mechanistic multicompartment permeability limited PBPK lung model has been previously defined in the evaluation of tuberculosis drugs.^30^ Briefly, the lung is described by seven segments (upper airway, lower airway, two left lung lobes, and three right lung lobes), each of which is further divided into four compartments: pulmonary capillary blood; tissue mass (pulmonary epithelial cells, smooth muscle cells, alveolar type I/II cells, macrophages, T‐cells, fibroblasts, extracellular matrix, vascular endothelial cells); fluid (mucus and epithelial lining fluid [ELF]); and alveoli air. The model incorporates the normal blood flow and airflow movements within the cardio‐pulmonary system. It assumes that each compartment is homogeneous, and that physiological and pharmacological parameters are constant, including blood flow and ventilation rates. For this analysis, we used data for the right lower lung lobe as a surrogate for lung tissue predicted PK, consistent with a previous study of PBPK modeling for COVID‐19.^27^ The compartment volumes were set as per the published methods, that is, fluid 25 mL, mass 470 mL, and blood 90 mL, and the default pH was 6.7 in the lung mass, 6.6 in ELF, and 7.4 in blood, based on healthy individuals.^30^ The predicted concentration of unbound pyronaridine or amodiaquine in lung mass was calculated by multiplying the total drug concentration in lung mass by the predicted fraction unbound in pulmonary mass (fu_mass_).

A perfusion‐limited model was used for DEAQ because this is a metabolite and, therefore, cannot be evaluated in the permeability‐limited PBPK model.^31^ In this case, tissue distribution was perfusion‐limited and the lung tissue was represented by an additional tissue compartment, rather than the default lung organ.^31^

### Evaluation of the efficacy of antimalarials for SARS‐Cov‐2

**FIGURE 1:**
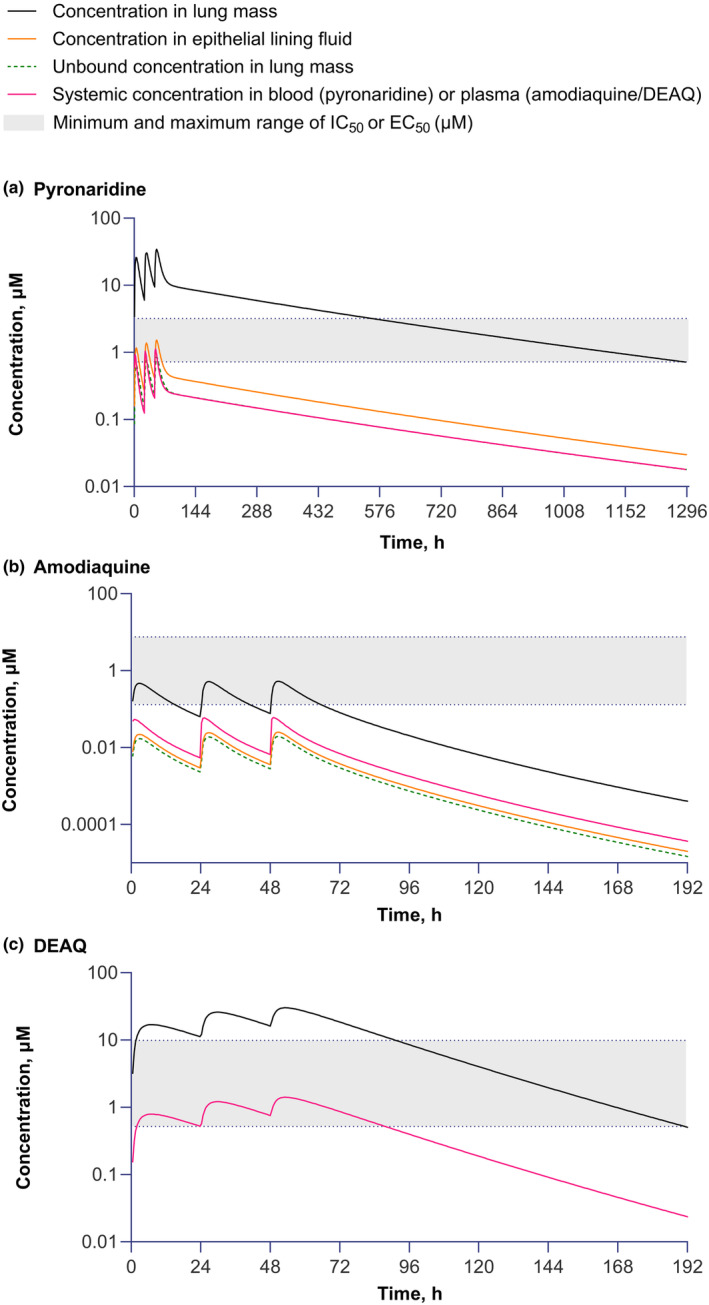
Simulated total drug concentrations in the healthy population for lung mass and epithelial lining fluid, unbound concentrations in lung mass, and total concentrations in blood or plasma for (a) pyronaridine using the permeability limited PBPK model; (b) amodiaquine using the permeability limited PBPK model; and (c) N‐desethylamodiaquine (DEAQ) using the perfusion‐limited PBPK model.

**FIGURE 2:**
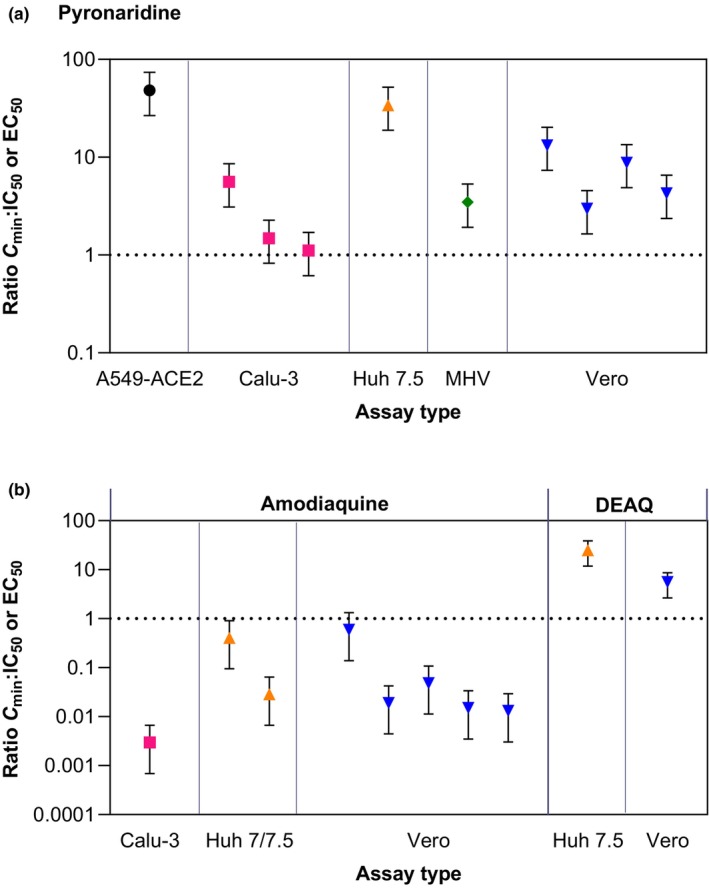
Assessment of predicted lung drug concentrations in the healthy population compared to in vitro activity. Ratio of predicted Day 3 trough concentrations in the lung mass relative to the respective reported EC/IC_50_ values against SARS‐CoV‐2 (Table 2) for (a) pyronaridine; and (b) amodiaquine and N‐desethylamodiaquine (DEAQ). Values are mean and whiskers are the 5th and 95th percentiles.

To collate the EC_50_ and IC_50_ values available for pyronaridine, amodiaquine, and DEAQ against SARS‐CoV‐2, a literature search was conducted on PubMed (23 June 2023) using the terms: pyronaridine OR amodiaquine OR DEAQ AND SARS‐CoV‐2 AND in vitro. The resulting 17 unique citations were examined for relevant data (eight articles) and any additional references cited in the publications were also examined. Antiviral activity varied considerably depending on the cell line and experimental conditions.^5^, ^6^, ^7^, ^8^, ^9^, ^13^, ^15^, ^22^, ^23^, ^24^, ^25^ As there is no consensus on the most clinically relevant assay,^32^ the minimum and maximum total IC_50_/EC_50_ values available for Vero cells were used to visually compare predicted total drug concentrations (Figure 1). Additionally, the ratio of the total trough concentration in the lung tissue pre‐dose on Day 3 (*C*_min_) versus the EC_50_ or IC_50_ values for each drug was calculated (Figure 2). Note that, as it is very difficult to estimate the free EC_50_ or IC_50_ based on the available data, we only evaluated and compared total concentrations. A ratio >1 for the Day 3 *C*_min_ relative to the EC_50_ or IC_50_ was considered to have the potential for clinical efficacy.

### Evaluation of COVID‐19 disease state

The conditions of patients infected with COVID‐19 were also considered. PBPK modeling has suggested that changes in lung pH during COVID‐19 infection can increase lung exposures to chloroquine, hydroxychloroquine, and azithromycin.^27^ Thus, an additional analysis repeated the permeability‐limited model for pyronaridine and amodiaquine but using a pH of 6, consistent with a diseased lung.^27^ Furthermore, COVID‐19 can cause changes in plasma proteins, with upregulation of alpha 1‐acid glycoprotein (AAG) and a reduction of human serum albumin (HSA) concentrations.^33^, ^34^ Pyronaridine was assumed to mainly be bound to AAG and amodiaquine to HSA. Thus, an analysis was conducted using both the decreased pH of 6 and plasma levels of 1.695 g/dL and 40 g/dL for AAG and HSA, respectively, consistent with the values reported from COVID‐19 patients.^33^, ^34^ These two modified analyses could not be done for DEAQ, because as a metabolite, these parameters cannot be varied in the perfusion‐limited PBPK model, though DEAQ is mainly bound to AAG.

## RESULTS

### PBPK model application: simulation of lung concentrations

A full PBPK model including a permeability‐limited lung compartment was used to provide a prospective prediction of pyronaridine and amodiaquine total and unbound concentrations in the lung.^29^, ^30^ A perfusion‐limited model was similarly used for the metabolite DEAQ.^31^ Artesunate concentrations were not evaluated as this drug does not show in vitro antiviral activity against SARS‐CoV‐2. Simulated drug concentrations in the different compartments are summarized in Table 1. The lung‐to‐plasma partition coefficient ratios for pyronaridine, amodiaquine, and DEAQ were 58, 11, and 21, respectively. Table 2 provides the range of EC_50_/IC_50_ for pyronaridine, amodiaquine, and DEAQ against SARS‐CoV‐2 across the reported in vitro experiments.^5^, ^6^, ^7^, ^8^, ^9^, ^13^, ^15^, ^22^, ^23^, ^24^, ^25^

**TABLE 1:** Predicted blood or plasma and lung concentrations.

Compartment	Healthy volunteers	
	*C*_max_, μM	*C*_min_, μM
*Pyronaridine*		
Lung mass (total)	34.2 (18.7, 54.4)	9.54 (5.28, 14.6)
ELF	1.52 (0.645, 2.55)	0.416 (0.214, 0.681)
Lung mass (UB)	0.856 (0.469, 1.36)	0.239 (0.132, 0.365)
Blood	1.12 (0.768, 1.57)	0.211 (0.115, 0.327)
L:B ratio (total)	30.5	45.2
L:B ratio (UB)	0.764	1.13
*Amodiaquine*		
Lung mass (total)	0.530 (0.122, 1.30)	0.0771 (0.0180, 0.173)
ELF	0.0250 (0.00553, 0.0597)	0.00360 (0.000871, 0.00853)
Lung mass (UB)	0.0194 (0.0476, 0.00446)	0.00282 (0.000659, 0.00633)
Plasma	0.0600 (0.0144, 0.142)	0.00660 (0.00146, 0.0153)
L:P ratio (total)	8.83	11.68
L:P ratio (UB)	0.323	0.427
*DEAQ*		
Mean lung mass	30.2 (16.0, 46.3)	16.2 (7.66, 25.0)
Mean plasma	1.41 (0.751, 2.17)	0.757 (0.360, 1.17)
L:P ratio	21.4	21.4

*Note*: *C*_max_ values are for Day 3 (highest value after 48 h) and *C*_min_ values are pre‐dose for the third dose on Day 3 (lowest value between 24 and 48 h) for pyronaridine and amodiaquine in the permeability‐limited PBPK model and for N‐desethylamodiaquine (DEAQ) in the perfusion‐limited PBPK model. Values are means (5th percentile, 95th percentile).

Abbreviations: ELF, epithelial lining fluid; L:B, lung‐to‐blood ratio; L:P, lung‐to‐plasma ratio; UB, unbound.

**TABLE 2:** In vitro activity reported for pyronaridine, amodiaquine, and N‐desethylamodiaquine (DEAQ).^5^, ^6^, ^7^, ^8^, ^9^, ^13^, ^15^, ^22^

References	Assay	Pyronaridine	Amodiaquine	DEAQ
Weston et al.^5^	Vero E6, IC_50_ (μM) MOI 0.004–0.01	–	2.59–4.94	–
Jeon et al.^6^	Vero, IC_50_ (μM) MOI 0.0125	–	5.15	–
Bocci et al.^7^	Vero E6, EC_50_ (μM)	–	0.13	–
Gendrot et al.^8^	Vero E6, EC_50_ (μM) MOI 0.25	0.72 ± 0.6	–	0.52 ± 0.2
	Vero E6, EC_90_ (μM) MOI 0.25	0.75 ± 0.4	–	1.93 ± 1.0
	Calu‐3, IC_50_ (μM)	1.7	26.1	>40
Bae et al.^9^	Calu‐3, IC_50_ (μM) (24 hpi) MOI 0.1	6.413	–	–
Puhl et al.^13^	Vero 76, EC_50_ (μM)	Not active	–	–
	Vero 6, EC_50_ (μM)	Not active	–	–
	Caco‐2, EC_90_ (μM)	5.49	–	–
	Calu‐3, IC_50_ (μM) MOI 0.5	Not active	–	–
	MHV, IC_50_ (μM)	2.75	–	–
	A549‐ACE2, IC_50_ (μM)	0.198	–	–
Dittmar et al.^15^	Huh 7.5, IC_50_ (μM)	0.28	0.19	0.65
	Vero, IC_50_ (μM)	3.2	1.6	2.9
	Calu‐3, IC_50_ (μM) (48 hpi) MOI 0.1	8.577	–	–
	Vero, IC_50_ (μM) (24 hpi) MOI 0.01	1.084	–	–
	Vero, IC_50_ (μM) (48 hpi) MOI 0.01	2.235	–	–
Si et al.^22^	Vero E6, IC_50_ (μM) MOI 0.1	–	7.5 ± 4.5	9.9 ± 4.1
Ko et al.^23^	Calu‐3, IC_50_ (μM) (24 hpi) MOI 0.1	–	>50	–
Zaliani et al.^24^	Vero E6, IC_50_ (μM) MOI 0.001	–	4.07	–
Persoons et al.^25^	Vero E6, EC_50_ (μM)		5.9 ± 1.2	
	Huh‐7, EC_50_ (μM)		2.7 ± 0.5	

Abbreviations: MOI, multiplicity of infection; hpi, hours post‐infection.

For pyronaridine, in the permeability‐limited lung PBPK model, the total predicted *C*_max_ in lung mass (34.2 μM) on Day 3 was 30.5‐fold greater than in blood (1.12 μM) (Figure 1a, Table 1). Similarly, the total *C*_min_ (corresponding to the time before administering the third dose) was 45.2‐fold greater in lung mass (9.54 μM) compared with blood (0.211 μM). Simulated pyronaridine concentrations in the lung mass exceeded the upper range of EC_50_/IC_50_ values for pyronaridine against SARS‐CoV‐2 reported for Vero cells (3.2 μM) until 557.3 h (~23 days) after the first dose and exceeded the lower range (0.72 μM) until 1290.8 h (~53 days) (Figure 1a). Predicted pyronaridine concentrations in the ELF also exceeded blood concentrations, but the difference was far less pronounced than for lung mass (Table 1). Predicted concentrations for unbound pyronaridine in lung mass were similar to the total plasma concentrations (Table 1).

For amodiaquine, although predicted total *C*_max_ in lung mass at Day 3 (0.530 μM) was 8.83‐fold greater than in plasma (0.060 μM) (Table 1), it did not exceed the upper limit of EC_50_/IC_50_ values for amodiaquine against SARS‐CoV‐2 reported for Vero cells (7.5 μM) (Figure 1b). Also, *C*_min_ values were below the lower limit of EC_50_/IC_50_ values (0.13 μM) (Figure 1b).

As the multi‐compartment permeability‐limited PBPK model cannot be applied to metabolites, a perfusion‐limited model was used for DEAQ.^31^ In the perfusion‐limited PBPK model, DEAQ predicted *C*_max_ on Day 3 in lung mass (30.2 μM) was 21.4‐fold greater than for plasma (1.41 μM) (Table 1). DEAQ simulated lung mass concentrations exceeded the upper limit of EC_50_/IC_50_ values for DEAQ against SARS‐CoV‐2 reported for Vero cells (9.9 μM) for DEAQ until 91.3 h (~4 days) after dosing and exceeded the lower limit (0.52 μM) for 190.9 h, (~8 days) (Figure 1c).

### Evaluation of efficacy potential against SARS‐CoV‐2

The simulated lung concentration profiles from the healthy population were then assessed relative to the in vitro activity against SARS‐CoV‐2 for pyronaridine, amodiaquine, and DEAQ (Table 2). For pyronaridine, the ratio of predicted trough concentration pre‐dose on Day 3 (*C*_min_) to the 50% effective (EC_50_) and 50% inhibitory (IC_50_) concentrations values reported for pyronaridine in vitro activity against SARS‐CoV‐2 exceeded the target of 1 for all assays (Figure 2a), meeting our pre‐defined criteria for potential efficacy in COVID‐19. The available data for DEAQ also indicated potential efficacy with values well above the target ratio of 1 (Figure 2b). However, for amodiaquine, the corresponding ratio for all assays was <1 (Figure 2b).

### Evaluation of COVID‐19 disease state

Three additional analyses were conducted using the permeability‐limited model to estimate the effect on pyronaridine and amodiaquine concentrations of key parameters known to be modified during COVID‐19 infection.^27^, ^33^, ^34^ With a decreased pH in the lung (pH 6), as observed in COVID‐19 infection, there was enhanced accumulation in the lung tissue with pyronaridine versus blood, and amodiaquine relative to plasma compared with the healthy lung (Table S1, Figures S1 and S2). The effect of modifying AAG and HSA was minor (data not shown). However, decreased pH plus modified AAG and HSA increased the lung exposures of pyronaridine and amodiaquine versus the peripheral compartment compared with the healthy population simulations (Table S1, Figures S3 and S4), though to a lesser extent than when pH 6 was considered alone. Therefore, the healthy population generated the most conservative predictions of lung exposures for pyronaridine and amodiaquine.

## DISCUSSION

This study aimed to explore the repurposing potential for the two approved antimalarial combination therapies, pyronaridine‐artesunate and artesunate‐amodiaquine, for the treatment of COVID‐19. PBPK modeling was used to simulate concentrations of pyronaridine, amodiaquine, and DEAQ in the lung tissue mass, ELF, and blood or plasma and compare these to the reported in vitro activity of the drugs against SARS‐CoV‐2. In this model, pyronaridine predicted *C*_max_ and *C*_min_ values on Day 3 in the lung mass were substantially higher than for pyronaridine in blood. Similarly, predicted DEAQ *C*_max_ and *C*_min_ values on Day 3 in the lung mass were higher than for DEAQ in plasma. Drug concentrations were maintained above the upper values for in vitro activity in Vero cells for pyronaridine for 557.3 h and for DEAQ for 91.3 h. Although amodiaquine has more potent in vitro activity than DEAQ against SARS‐CoV‐2 (Table 1), lung exposures were not greater than the in vitro activity in Vero cells. However, as exposures of the metabolite are significantly greater than the parent (about 10‐fold in terms of total exposures and 100‐fold if considering the free concentrations), it is mainly DEAQ which contributes to the potential efficacy of amodiaquine.

In this analysis, the lung was considered the main target organ for pharmacological disruption of SARS‐CoV‐2 infection. Even though ex vivo experiments have shown that Omicron (B.1.1.529) replicates more rapidly in bronchi and more slowly in lung parenchyma than wild‐type virus or the Alpha (B.1.1.7), Beta (B.1.351), and Delta (B.1.617.2) variants,^35^ the involvement of the lung is still a critical driver of COVID‐19 pathophysiology, with type II pneumocytes most affected in COVID‐19‐related deaths.^36^ Thus, attenuation of the replicative capacity of the virus in this organ remains a target for reducing disease progression. Once infection is established, the virus can target other organs.^37^ Therefore, it may be necessary to consider drug exposure in a range of target organs when optimizing COVID‐19 pharmacotherapies.

Pyronaridine and amodiaquine were evaluated in the PBPK model at the approved doses in the marketed combinations of pyronaridine‐artesunate and artesunate‐amodiaquine.^10^, ^18^ One of the advantages of drug repurposing that can accelerate drug development is the known pharmacokinetic, safety, and tolerability profile in the licensed indication and the availability of the marketed product.^15^, ^32^ Although the new target population may differ, the pre‐clinical safety profile is transferrable, and the chances of success are higher than with the evaluation of new chemical entities. Even though drug combinations can be considered at an early stage of development, the activity and effective dose of each of the components of any combination must be demonstrated. Thus, initial PBPK simulations, as reported here, should consider single agents at the doses for which they are licensed. Dose optimization and potential novel drug combinations can be subsequently investigated.

The PBPK models for pyronaridine and amodiaquine have been previously verified against clinical pharmacokinetic data in healthy volunteers using blood and plasma concentrations, respectively.^29^ However, at the time of this analysis, there were no published pharmacokinetic data for pyronaridine, amodiaquine, or DEAQ in ELF or lung tissue in COVID‐19 patients from clinical studies. In terms of preclinical models, although pyronaridine had been evaluated in a humanized mouse model of COVID‐19 infection, no pharmacokinetic data were reported in that study.^16^ However, a recent evaluation of pyronaridine‐artesunate pharmacokinetics in hamsters, including the development of a minimal PBPK model, indicated AUC lung to AUC blood ratios for pyronaridine of 24.5 on Day 1 and 51.0 on Day 3, supporting the preferential distribution of the drug into the lung.^38^

The limitations of this study are related to the assumptions around the in vitro data, the absence of a verified ‘COVID‐19’ virtual population, and the lack of clinical data for pyronaridine and amodiaquine in COVID‐19 for model verification, as discussed below.

In terms of the in vitro data, the range of in vitro activity against SARS‐CoV‐2 demonstrated by pyronaridine, amodiaquine, and DEAQ in the different cell lines is highlighted. It remains unclear which of the in vitro assays is the most clinically relevant for the treatment of COVID‐19. Also, experimental conditions vary, and the information needed to estimate free EC_50_/IC_50_ values is not available. For this reason, the comparison was only made using total concentrations. We assumed a ratio of drug concentration to EC_50_/IC_50_ against SARS‐CoV‐2 of >1 as a minimum criterion to select drugs for clinical investigation, but there is no clinical validation for this cutoff. Although EC_90_ is a more stringent cutoff for assessing antiviral activity, given the uncertainty around the in vitro data, the strategy at that time was to not rule out drugs that may have some efficacy and so the EC_50_/IC_50_ was used. More recently, the hollow fiber infection model (HIFM) has been applied to evaluate potential drugs for SARS‐CoV‐2.^39^, ^40^ This two‐compartment model allows drug pharmacodynamics to be evaluated over a range of drug disposition profiles and pathogen loads and can incorporate different cell types relevant to the disease pathology, such as primary human epithelial cells or primary human lung cells. For example, HIFM was used to investigate why molnupiravir had no clinical benefit in hospitalized COVID‐19 patients, despite demonstrating good in vitro activity in cell lines.^40^ Although the model has not been used so far to evaluate pyronaridine or amodiaquine against SARS‐CoV‐2, it could be a useful approach for drug repurposing programs to evaluate anti‐infective activity and investigate optimized dosing strategies.^39^ Moreover, despite the cost of such experiments, they would be technically feasible to conduct during a public health emergency similar to the COVID‐19 pandemic.

As there is no validated ‘COVID‐19’ virtual population, we performed the simulations in the healthy population, and this generated the most conservative predicted lung concentrations, when compared with simulations performed with adjusted pH and plasma protein concentrations. Thus, the simulations reported here did not consider the entirety of potential physiological changes in COVID‐19 patients that may affect drug pharmacokinetics, or patients with co‐morbidities known to be associated with pharmacokinetic variability, such as organ impairment.^41^, ^42^, ^43^ PBPK modeling has suggested that changes in lung pH during COVID‐19 infection can increase lung exposures to chloroquine, hydroxychloroquine, and azithromycin.^27^ Additional simulations run with a lower lung pH of 6 to reflect the COVID‐infected lung,^27^ instead of pH 6.7 as in the healthy lung, predicted higher lung concentrations for pyronaridine and amodiaquine. This provides further support for these drugs as candidates for repurposing in COVID‐19. Clinical observations indicate that serum concentrations of AAG are elevated in COVID‐19 patients,^33^ whereas HSA levels have been shown to decline.^34^ Incorporating these modifications into the PBPK model using a virtual population with AAG and HSA levels corresponding to those reported in the literature showed increased lung exposures of both pyronaridine and amodiaquine versus the healthy population but with some attenuation versus the simulations using a pH of 6. Thus, lung exposures were far more sensitive to changes in pH than plasma proteins. However, notably, for an inflammatory condition such as COVID‐19, the potential effects of drugs on inflammatory processes and the contribution of immunological responses to clinical outcomes should be investigated clinically.

Ideally, a PBPK model would be verified using clinical PK data for the investigated drugs from patients with the disease under consideration. One clinical study evaluating pyronaridine‐artesunate and artesunate‐amodiaquine in COVID‐19 outpatients versus standard‐of‐care in South Africa has been published.^44^ However, given the difficult circumstances under which the study was conducted at the height of the pandemic, blood samples were limited, and the full pharmacokinetic profile for pyronaridine in COVID‐19 patients could not be determined. Clinical outcomes for this trial were inconclusive, possibly because the patient population had ‘mild’ illness, a low risk of disease progression, and 29% had positive baseline SARS‐CoV‐2 serology findings, indicating prior exposure to the virus.^44^ Larger clinical studies are ongoing for pyronaridine‐artesunate (ClinicalTrials.gov ID: NCT04475107; NCT04701606; NCT05084911) and artesunate‐amodiaquine was included in the ANTICOV adaptive platform study.^45^ Thus, should these trials be completed, there may be the opportunity to clinically validate the predicted plasma concentrations versus the observed data from patients. This would allow refinement of the model by including physiological changes specific to COVID‐19 patients to inform further work, such as potential dose optimization required for these patients.

Although our PBPK strategy focused on drug repurposing specifically for COVID‐19, this general approach may have utility in other repurposing programs. This paper presents how this approach was used to identify potential new therapeutic agents for an emerging infectious disease during a public health emergency when access to clinical populations was limited and urgent solutions were required. During the COVID‐19 pandemic, PBPK modeling was applied to evaluate potential drug repurposing candidates, including the accumulation of drug into ELF and lung mass, similar to the current evaluation.^38^, ^42^, ^43^, ^46^, ^47^, ^48^, ^49^, ^50^ Notably, PBPK modeling findings were informative in the clinical development of remdesivir.^42^, ^50^

A key advantage of PBPK modeling is that it enables early simulation and prediction of drug behavior based on known physiological and biochemical parameters before clinical data become available. In the case of drug repurposing, clinical pharmacokinetic data are available from healthy adults for model verification increasing the confidence of the predictions. In particular, the model allows the estimation of drug concentrations at the target site, in this case, the lung. Additionally, PBPK models can simulate various ‘what‐if’ scenarios, predicting drug concentrations across different conditions, as was shown for changes in pH and protein binding in this study. It thereby provides an early assessment of whether a drug has plausible potential for repurposing to the new indication. Similar approaches using PBPK models to explore drug exposure in lung tissue have been applied to investigate new therapies for tuberculosis.^30^, ^48^ Once clinical data in the new indication become available, the PBPK model can be refined. Furthermore, unlike empirical pharmacokinetic/pharmacodynamic (PKPD) models, PBPK models can extrapolate beyond the initial study population, providing insights into how a drug might perform in diverse patient groups, such as children, pregnant women, or those with renal or hepatic impairment. By estimating local drug concentrations at the site of action and accounting for inter‐subject variability, PBPK models facilitate a comprehensive evaluation of drug behavior, supporting informed decision‐making in the drug repurposing process.

In this study, we applied PBPK modeling to select candidates for clinical trials by simulating concentrations in the lung and comparing these to the available in vitro activity data against SARS‐CoV‐2. PBPK modeling indicated lung exposures of pyronaridine and DEAQ exceeded target concentrations following administration with the dose regimens currently approved for the treatment of uncomplicated malaria. These findings suggested potential utility for both pyronaridine‐artesunate and artesunate‐amodiaquine for the treatment of COVID‐19 and were used to plan ongoing clinical studies that are required to assess treatment efficacy.

## AUTHOR CONTRIBUTIONS

N.A. and N.R. wrote the manuscript. N.A., L.M.A., and J.J.B. designed the research. N.A., L.M.A., and J.J.B. performed the research. N.A., L.M.A., J.J.B., N.R., T.N.C.W., and J.J.M. analyzed the data.

## FUNDING INFORMATION

This study was funded in whole or in part by the Bill & Melinda Gates Foundation via MMV Medicines for Malaria Venture (Investment ID INV‐007155) and Certara UK Ltd (INV‐040110 and INV‐051204). The views expressed do not reflect the official views of the Bill & Melinda Gates Foundation. Under the grant conditions of the Foundation, a Creative Commons Attribution 4.0 Generic License has already been assigned to the Author Accepted Manuscript version that might arise from this submission. The study was also supported by Medicines for Malaria Venture with grants obtained from UK Aid from the UK Foreign, Commonwealth and Development Office under the provision of the COVID‐19 Therapeutics Accelerator in partnership with Wellcome, the Bill and Melinda Gates Foundation, and Mastercard.

## CONFLICT OF INTEREST STATEMENT

N.A., T.N.C.W., and J.J.M. are employees of MMV Medicines for Malaria Venture. L.A. and J.J.B. are employees of Simcyp and may hold stocks or shares in the company. N.R. is an employee of Magenta Communications Ltd which was funded by MMV Medicines for Malaria Venture.

## Supporting information


Appendix S1.

## Data Availability

Compound files are freely available at: https://pbpkrepository.certara.co.uk
