# Supplementary material for: PBPK‐led assessment of antimalarial drugs as candidates for Covid‐19: Simulating concentrations at the site of action to inform repurposing strategies
Source: Clin Transl Sci. 2024 Jul 17;17(7):e13865. doi: 10.1111/cts.13865 (PMC11254780; doi:10.1111/cts.13865)

Supplemental material

PBPK-led assessment of antimalarial drugs as candidates for Covid-19: simulating concentrations at the site of action to inform repurposing strategies

Nada Abla, et al.

TABLE S1. Analysis of adjusted pH and plasma proteins based on COVID-19 disease state. Predicted blood or plasma and lung concentrations for pyronaridine and amodiaquine in the permeability-limited PBPK model. 2

FIGURE S1. Analysis at lung pH observed in COVID-19 patients (pH 6). Simulated total concentrations in lung mass and epithelial lining fluid, unbound concentrations in lung mass, and total concentrations in blood or plasma for A) pyronaridine using the permeability limited PBPK model; and B) amodiaquine using the permeability limited PBPK model. 3

FIGURE S2. Analysis at lung pH observed in COVID-19 patients (pH 6). Ratio of predicted Day 3 trough concentrations in the lung mass relative to the respective reported EC/IC_{50} values against SARS-CoV-2 (Table 2) for A) pyronaridine; and B) amodiaquine. 4

FIGURE S3. Analysis at lung pH observed in COVID-19 patients (pH 6), plus plasma proteins adjusted based on values from COVID-19 patients for alpha 1-acid glycoprotein (1.695 g/dL), and human serum albumin (40 g/dL). Simulated total concentrations in lung mass and epithelial lining fluid, unbound concentrations in lung mass, and total concentrations in blood or plasma for A) pyronaridine using the permeability limited PBPK model; and B) amodiaquine using the permeability limited PBPK model. 5

FIGURE S4. Analysis at lung pH observed in COVID-19 patients (pH 6), plus plasma proteins adjusted based on values from COVID-19 patients for alpha 1-acid glycoprotein (1.695 g/dL), and human serum albumin (40 g/dL). Ratio of predicted Day 3 trough concentrations in the lung mass relative to the respective reported EC/IC_{50} values against SARS-CoV-2 (Table 2) for A) pyronaridine; and B) amodiaquine. 6

TABLE S1. Analysis of adjusted pH and plasma proteins based on COVID-19 disease state. Predicted blood or plasma and lung concentrations for pyronaridine and amodiaquine in the permeability-limited PBPK model.

**Compartment**	**Diseased lung pH[a]**		**Diseased lung pH, AAG, HSA[a]**	
**Pyronaridine**	C_{max}**, µM**	C_{min}**, µM**	C_{max}**, µM**	C_{min}**, µM**
Lung mass (total)	309 (115, 558)	213 (77.5, 386.5)	257 (114,435)	79.2 (21.8, 167)
ELF	8.80 (2.35, 17.5)	6.05 (1.59, 12.3)	3.26 (1.08, 5.87)	1.03 (0.238, 2.43)
Lung mass (UB)	7.75 (2.88, 14.0)	5.33 (1.94. 9.69)	6.45 (2.87, 10.9)	1.99 (0.546, 4.19)
Blood	1.11 (0.767, 1.55)	0.193 (0.104, 0.298)	1.23 (0.848, 1.76)	0.269 (0.157, 0.398)
L:B ratio (total)	278	1104	209	294
L:B ratio (UB)	6.98	27.6	5.24	7.40
**Amodiaquine**	C_{max}**, µM**	C_{min}**, µM**	C_{max}**, µM**	C_{min}**, µM**
Lung mass (total)	2.68 (0.620, 6.09)	1.15 (0.239, 2.63)	2.41 (0.542, 5.63)	0.949 (0.218, 2.09)
ELF	0.109 (0.0256, 0.237)	0.046 (0.0110, 0.101)	0.112 (0.0264, 0.247)	0.0440 (0.0103, 0.0968)
Lung mass (UB)	0.111 (0.0252, 0.257)	0.0477 (0.0991, 0.109)	0.0811 (0.0182, 0.189)	0.0319 (0.00732, 0.0703)
Plasma	0.0599 (0.0144, 0.142)	0.00664 (0.00146, 0.0156)	0.0544 (0.0126, 0.128)	0.00593 (0.00134, 0.0140)
L:P ratio (total)	44.7	164	44.3	160
L:P ratio (UB)	1.85	6.86	1.49	5.38

C_{max} values are for Day 3 (highest value after 48 h) and C_{min} values are pre-dose for the third dose on Day 3 (lowest value between 24 and 48 h) for pyronaridine and amodiaquine in the permeability-limited PBPK model. Values are means (5th percentile, 95th percentile). ELF, epithelial lining fluid; L:B, lung-to-blood ratio; L:P, lung-to-plasma ratio; UB, unbound.

[a] Altered physiological parameters with COVID-19 were lung pH of 6, plasma levels of alpha 1-acid glycoprotein (AAG) of 1.695 g/dL, and plasma levels of human serum albumin (HSA) was 40 g/dL. As a metabolite, the parameters cannot be varied for N-desethylamodiaquine (DEAQ) in the perfusion-limited PBPK model.

FIGURE S1. Analysis at lung pH observed in COVID-19 patients (pH 6). Simulated total concentrations in lung mass and epithelial lining fluid, unbound concentrations in lung mass, and total concentrations in blood or plasma for A) pyronaridine using the permeability limited PBPK model; and B) amodiaquine using the permeability limited PBPK model.

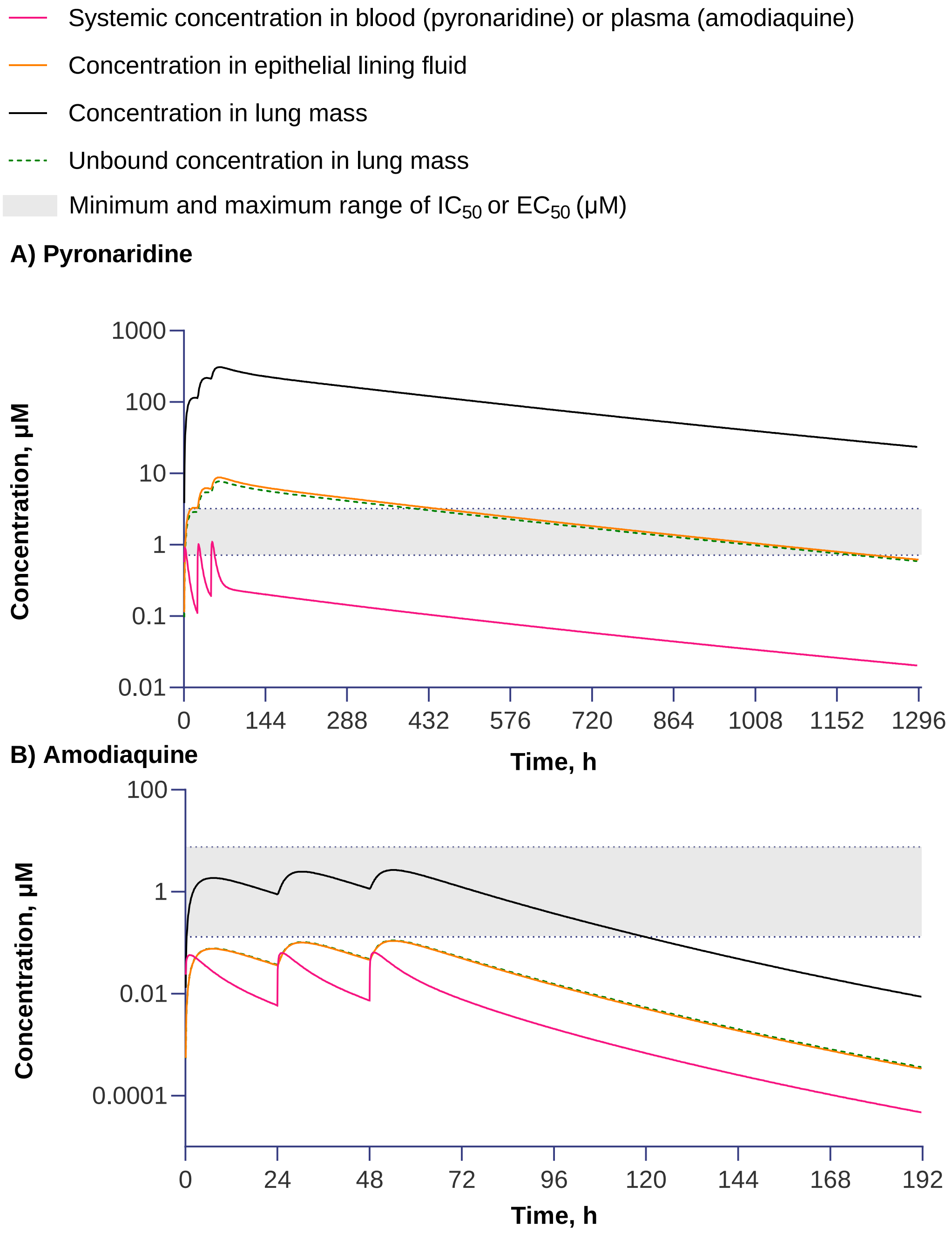

FIGURE S2. Analysis at lung pH observed in COVID-19 patients (pH 6). Ratio of predicted Day 3 trough concentrations in the lung mass relative to the respective reported EC/IC_{50} values against SARS-CoV-2 (Table 2) for A) pyronaridine; and B) amodiaquine.

Values are mean and whiskers are the 5th and 95th percentiles.

A) Pyronaridine

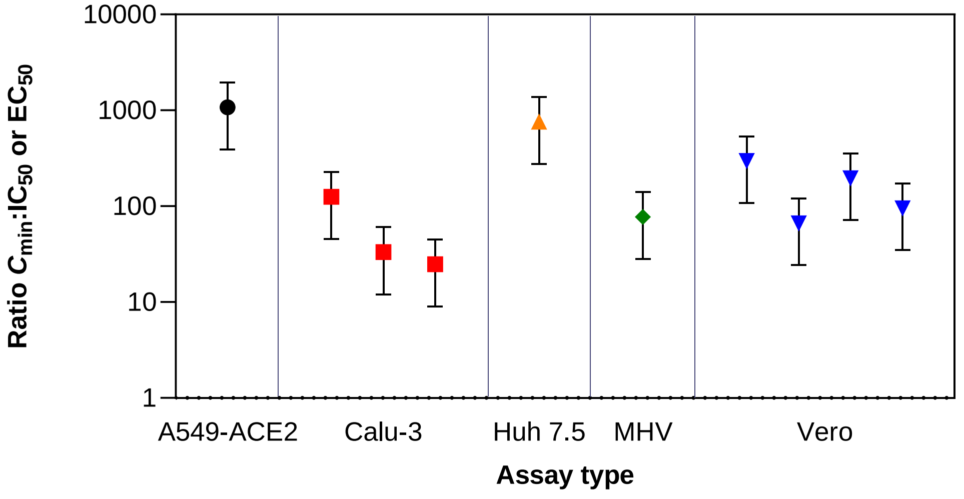

B) Amodiaquine

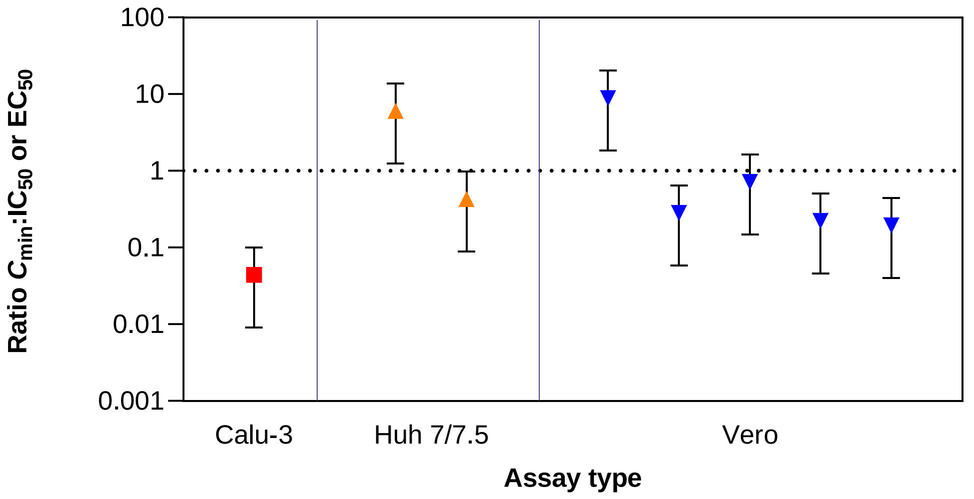

FIGURE S3. Analysis at lung pH observed in COVID-19 patients (pH 6), plus plasma proteins adjusted based on values from COVID-19 patients for alpha 1-acid glycoprotein (1.695 g/dL), and human serum albumin (40 g/dL). Simulated total concentrations in lung mass and epithelial lining fluid, unbound concentrations in lung mass, and total concentrations in blood or plasma for A) pyronaridine using the permeability limited PBPK model; and B) amodiaquine using the permeability limited PBPK model.

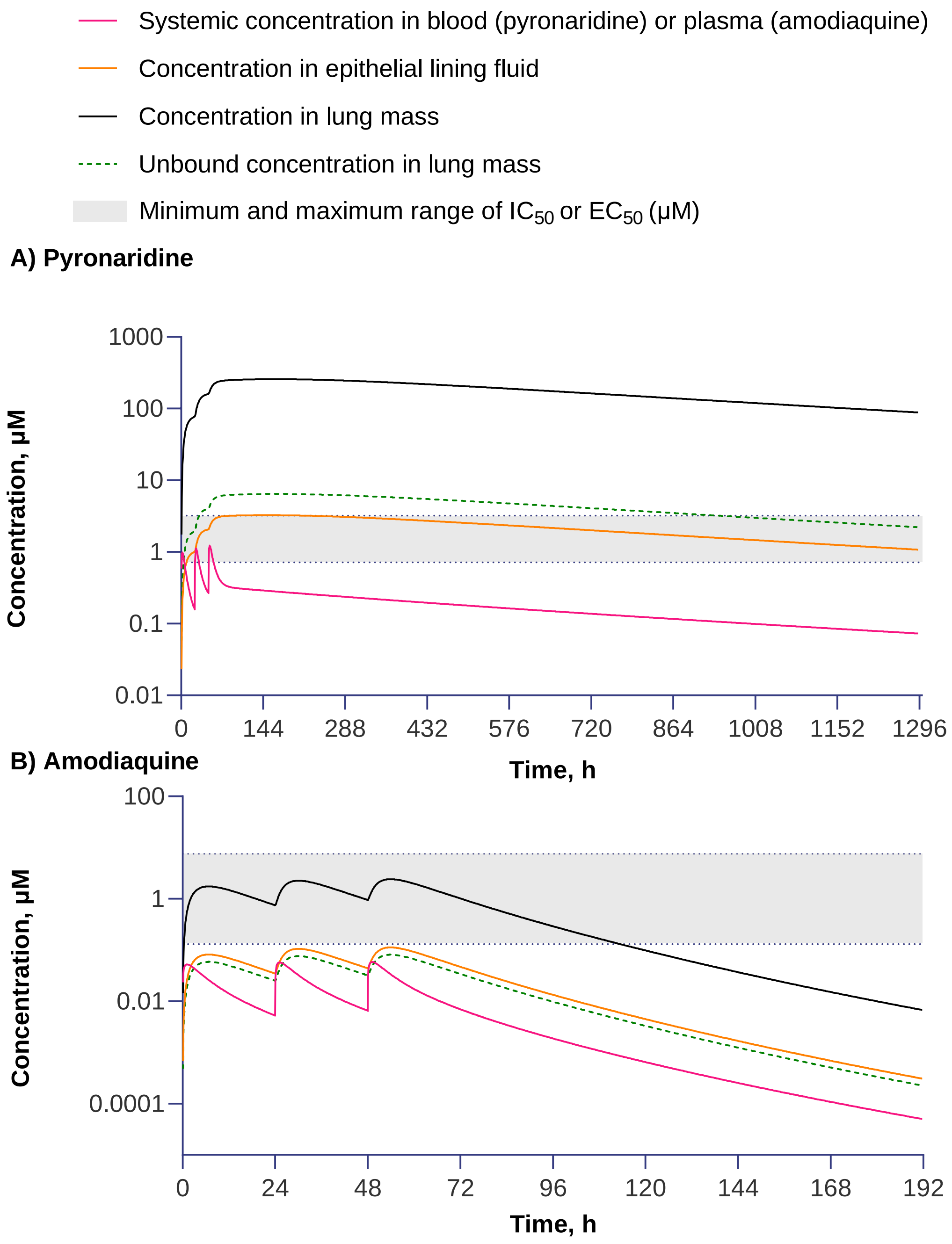

FIGURE S4. Analysis at lung pH observed in COVID-19 patients (pH 6), plus plasma proteins adjusted based on values from COVID-19 patients for alpha 1-acid glycoprotein (1.695 g/dL), and human serum albumin (40 g/dL). Ratio of predicted Day 3 trough concentrations in the lung mass relative to the respective reported EC/IC_{50} values against SARS-CoV-2 (Table 2) for A) pyronaridine; and B) amodiaquine.

Values are mean and whiskers are the 5th and 95th percentiles.

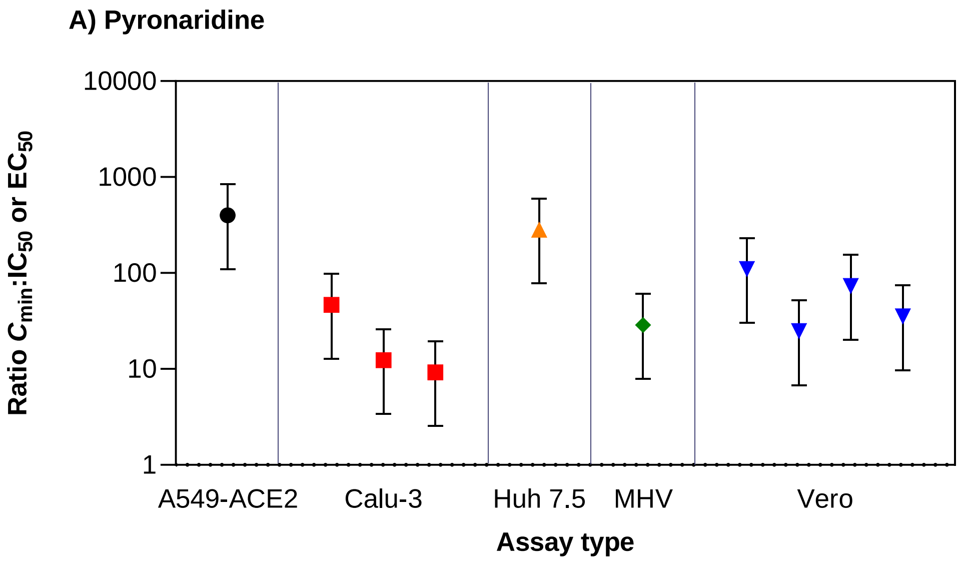

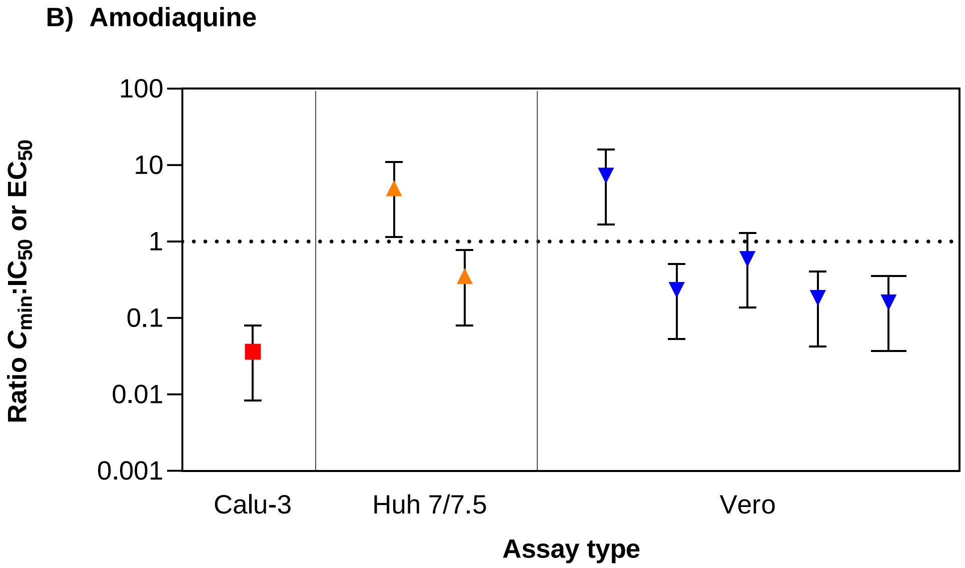

Supplement: Supplementary file 1 — Appendix S1. [file CTS-17-e13865-s001.pdf]
